# Early Posttreatment Audiometry Underestimates Hearing Recovery after Intratympanic Steroid Treatment of Sudden Sensorineural Hearing Loss

**DOI:** 10.1155/2011/465831

**Published:** 2011-11-28

**Authors:** Benjamin J. Wycherly, Jared J. Thompkins, H. Jeffrey Kim

**Affiliations:** ^1^Department of Otolaryngology-Head and Neck Surgery, Georgetown University Medical Center, 3800 Reservoir Road, NW, Washington, DC 20007, USA; ^2^Georgetown University Medical Center, 3900 Reservoir Road, NW, Medical Dental Building, Washington, DC 20057, USA

## Abstract

*Objective*. To review our experience with intratympanic steroids (ITSs) for the treatment of idiopathic sudden sensorineural hearing loss (ISSNHL), emphasizing the ideal time to perform follow-up audiograms. *Methods*. Retrospective case review of patients diagnosed with ISSNHL treated with intratympanic methylprednisolone. Injections were repeated weekly with a total of 3 injections. Improvement was defined as an improved pure-tone average ≥20 dB or speech-discrimination score ≥20%. *Results*. Forty patients met the inclusion criteria with a recovery rate of 45% (18/40). A significantly increased response rate was found in patients having an audiogram >5 weeks after the first dose of ITS (9/13) over those tested ≤5 weeks after the first dose of ITS (9/27) (*P* = 0.03). *Conclusions*. Recovery from ISSNHL after ITS injections occurs more frequently >5 weeks after initiating ITS. This may be due to the natural history of sudden hearing loss or the prolonged effect of steroid in the inner ear.

## 1. Introduction

Sudden hearing loss is a devastating disease for patients and represents a true otologic emergency. The etiologies of sudden hearing loss are many, but only about 10–15% of cases have an identifiable cause [1]. Oral steroids have been effective in many patients and currently represent the standard treatment for idiopathic sudden sensorineural hearing loss (ISSNHL) [2–4].

Intratympanic steroids (ITSs) may treat ISSNHL more effectively than oral steroids [5–12]. Data to support this hypothesis are limited almost exclusively to case series, which differ in the type of steroid used, the steroid dose, the dose frequency, the method of injection, the existence of previous treatment with oral steroids, and the outcome measurements. In addition, these studies do not specifically address the timing of the posttreatment audiogram in determining treatment success. We reviewed our experience with ITS, which included an analysis of the timing of post-treatment audiograms. In doing so we sought to determine if there is a different recovery rate depending on when audiograms are performed after completing ITS treatment. Most cases of sudden hearing loss are idiopathic not because they do not have a cause but because we are unable to identify it. We presumed that any group of idiopathic hearing loss patients is a heterogeneous one with variable response to steroids and a variable time course to recovery including both early and late responders.

## 2. Materials and Methods

### 2.1. Inclusion Criteria

After obtaining institutional board approval, we performed a retrospective chart review of all patients undergoing ITS injection at our tertiary referral center from October 2001 to July 2008. To be included in the study, patients had to have a sensorineural hearing loss of at least 30 dB in 3 contiguous frequencies occurring in less than 72 hours. Additional inclusion and exclusion criteria are listed in Table 1. In all patients serologic studies included an erythrocyte sedimentation rate, Lyme titer, anti-nuclear antibody titer, rheumatoid factor, and a fluorescent treponemal antibody absorption (FTA-ABS) study.

### 2.2. Audiometric Measurements

Patients were evaluated for pure-tone thresholds and speech intelligibility both before and after steroid injection. The pure-tone average (PTA) was calculated as an average of thresholds measured at 0.5, 1.0, and 2.0 KHz. Speech-discrimination scores (SDSs) were calculated as the percent of phonetically balanced, monosyllabic words pronounced correctly.

### 2.3. Method of Intratympanic Injection

First, phenol was applied topically to anesthetize the posterior inferior quadrant of the tympanic membrane (the area overlying the round window niche). A single myringotomy was then performed with a disposable knife. Approximately 0.3 to 0.5 mL of 40 mg/mL methylprednisolone mixed 9 : 1 with 2% lidocaine was then delivered through the myringotomy via a 25 gauge spinal needle. The patients were then instructed to maintain their head turned 45° away from the injected ear in a reclined position for 30 minutes and were encouraged not to swallow if possible. Injections were repeated at approximately one-week intervals for a total of 3 injections (3 injections over approximately 15 days).

### 2.4. Statistical Analysis

Statistical analysis was performed using Microsoft Excel version 2002 (Redmond, WA) and SAS version 9.2 (SAS Institute Inc, Cary, North Carolina). Improvement was defined as an improved pure-tone average ≥20 dB or speech-discrimination score ≥20%. The variables evaluated included time to post-treatment audiogram, age, gender, sidedness of hearing loss, days from onset to ITS treatment (>14 days), prior treatment with oral steroids, presence of vertigo, and presence of tinnitus.

The primary covariate of interest was the time to post-treatment audiogram which was dichotomized at 5 weeks (35 days) after the first dose of ITS. Pre-ITS PTA and SDS were compared using paired *t*-tests. Since expected cell counts were greater than five for all covariates, chi-square tests were used to determine the association between categorical patient characteristics and improvement and *t*-tests were used for continuous characteristics. Logistic regression was used to estimate adjusted odds ratios and 95% confidence intervals for variables which were found to be significant. Variable selection for the model was based on the significance (at 0.05 level) of univariate associations with improvement.

## 3. Results

### 3.1. Patient Characteristics

During the study period, 172 series of injections were performed in 151 patients with 60 patients diagnosed with ISSNHL. Of those 60, 13 were excluded due to insufficient data, and 7 were excluded because they did not have at least a 30 dB hearing loss in 3 contiguous frequencies; therefore, a total of 40 patients were included in the review.

The demographic information for our patient population is displayed in Table 2. The average number of days from the onset of hearing loss to the initiation of ITS was 22.7, with a nearly equal distribution of patients starting treatment less than 2 weeks (15), between 2 to 4 weeks (12), and greater than 4 weeks (13) after the onset of hearing loss.

### 3.2. Rate and Degree of Recovery

The overall response rate was 45% (18/40). PTA decreased an average of 17 dB from pre- to post-treatment (95% CI: −24, −11; *P* < 0.001) and SDS increased an average of 12% (95% CI: 2, 21; *P* = 0.02; Table 3). Patients with serviceable hearing (defined as a SDS ≥50% and a PTA ≤50 dB) increased from 7/40 (17.5%) prior to ITS to 15/40 (37.5%) after ITS.

### 3.3. Patient Characteristics Associated with Improvement

**Figure 1:**
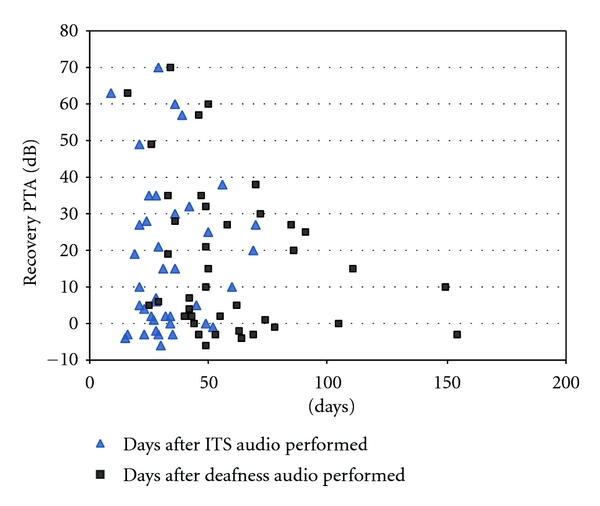
Scatterplot: time from deafness and time from intratympanic steroids (ITSs) to audiogram; hearing recovery in decibels (dBs).

**Figure 2:**
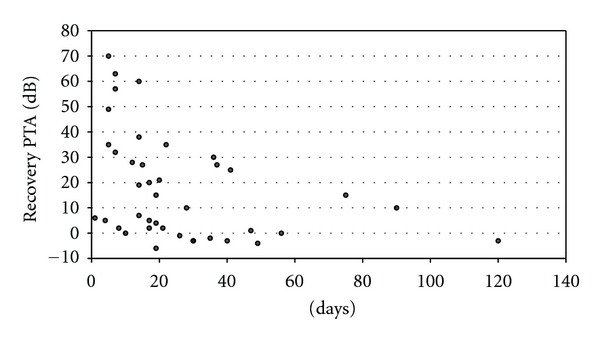
Scatterplot: duration of deafness prior to first dose of intratympanic steroids (ITSs) and degree of hearing recovery in decibels (dBs).

Overall patient characteristics and improvement status are shown in Table 4. The average number of days that an audiogram was performed was 33.2 days after the first steroid injection. Patients who had audiometric testing more than 35 days (5 weeks) after the first dose of ITS were more likely to demonstrate hearing improvement than those who received testing earlier (69% versus 33%; *P* = 0.03). The number of days from hearing loss to starting ITS was not significantly different between patients who had an audiogram at more than 5 weeks or less than 5 weeks after the first dose of ITS (*P* = 0.32). Regression analysis was performed to determine if either the days from hearing loss to the audiogram or the days from starting ITS to the audiogram were significant (Figure 1). Neither the days from hearing loss to audiogram (*r* ^2^ = 0.087, d.f. =39, *P* = 0.06) nor the days from ITS to audiogram (*r* ^2^ = 0.0005, d.f. = 39, *P* = 0.89) were significant, although the former demonstrated a trend toward a lesser likelihood of improvement with a longer duration of hearing loss to audiogram. Patients who received ITS ≤21 days after sudden hearing loss were more likely to improve than those who had a treatment delay of >22 days (50% versus 38%, *P* = 0.02); see also Figure 2.

Patients experiencing vertigo were more like to improve than patients who did not (65% versus 35%; *P* = 0.03). No other patient characteristics were significantly associated with improvement. After adjusting for vertigo, patients who had testing more than 5 weeks after the first dose of ITS were 6.7 times more likely to show improvment than patients who had earlier testing (95% CI: 1.3, 35; *P* = 0.02) and patients with vertigo were 6.2 times more likely to improve than patients who did not experience vertigo (95% CI: 1.3, 29; *P* = 0.02), as shown in Table 5. Patients with vertigo (12/40) started treatment an average of 18 days after the onset of hearing loss while patients who did not experience vertigo with their hearing loss started treatment an average of 32 days after the onset of hearing loss (*P* = 0.07, paired *t*-test).

### 3.4. Complications

One patient experienced tinnitus for several weeks after the first injection which resolved spontaneously. All patients tolerated the procedure well. There were no episodes of vertigo with the administration of the steroid. All tympanic membranes healed after completion of the treatment.

## 4. Discussion

Our data are similar to other reviews of ITS with regard to demographics of ISSNHL and response rate to ITS; however, our review differs in that it specifically addresses the timing of post-treatment audiograms when assessing treatment response. Most other reviews report post-treatment audiograms as taking place during a certain range of weeks or months after treatment has concluded, with all patients falling within that stated range.

A few studies have reported the use of serial audiograms during or immediately after the completion of treatment with ITS. These studies have found that the greatest treatment response is after the first injection. Guan-Min et al. [5] performed serial audiograms in patients who received intratympanic dexamethaxone after failure with oral steroids. Audiograms were performed after each of 3 injections. The greatest response to treatment was 7 days after the first injection with a steadily decreasing steroid effect thereafter. There were significant differences between the PTA after the first and third injection, corresponding to 7 and 21 days after treatment began. Choung et al. [13] also performed serial audiograms after intratympanic dexamethasone was administered twice a week for 2 weeks. Audiograms were performed after each injection and found a similar response early, with the greatest response in 35.8% of ears after the first injection, 14.7% after the second, and 11.8% after the third; however, Choung also performed an audiogram 1 week after the final injection. There was an increased response rate from 11.8% on day 14 to 20.6% on day 21. Whether or not this increased response represents a significant delayed response to treatment is uncertain. The studies of Guan-Min and Choung provide valuable information regarding the early response to treatment with ITS but do not address the response to treatment that may occur several weeks after the completion of treatment.

Additional studies have been published which note a more delayed response. Slattery et al. [14] reviewed their experience with oral steroids where audiograms were performed on average 44 days after hearing loss. A subset of those patients had an additional audiogram 4 months (average) after the onset of hearing loss for comparison. A statistically significant improvement in the PTA was found between the 44-day audiogram to the second post-treatment audiogram. Battaglia et al. [12] performed a double-blinded, placebo-controlled, randomized study with patients receiving either intratympanic dexamethasone and an oral placebo, high-dose oral steroids and intratympanic saline, or intratympanic dexamethasone and high-dose oral steroids. Treatment was administered once a week for 3 weeks with serial audiograms and an additional audiogram performed 4 weeks after the initial injection. Although not discussed in their paper, the data suggest an increased probability of improvement in all 3 groups when comparing the audiograms from immediately after treatment with those performed at least 4 weeks after the initial injection. A statistical analysis was not performed on this particular aspect of the data.

By definition, ISSNHL is from an unknown etiology and is a result of multiple unknown causes. Within this heterogeneous group, there are likely to be early, late, and nonresponders to steroid treatment. There may also be a number of patients who are recovering spontaneously, regardless of steroid therapy. This has been reported to be somewhere between 31% and 65% [2, 3, 15, 16]. Nevertheless, the timing of the audiogram will determine the observed response; therefore, when reporting results of idiopathic hearing loss, studies should, at the least, report when the audiograms were performed. Ideally, a series of audiograms should be administered during and after treatment to document the timing of treatment response. This will identify early versus late responders and may provide important clues to the etiology of hearing loss. It may also have implications with regard to the determination of the length of treatment.

Another possibility to explain the late effect of ITS is how long it may affect inner ear cells. Intratympanic methylprednisolone has been detected in the perilymph and endolymph in guinea pigs for a fairly short duration; it achieves its highest concentration at 1-2 hours and is present for at least 6 hours, but it is no longer detectable at 24 hours [17]. Despite the brief existence of steroids within the inner ear fluid, the effect may be more durable and has not yet been determined. Steroids effect gene expression in cochlear cells to alter both inflammatory processes [18] and ion homeostasis [19, 20]. A delayed effect of treatment may well be via a mechanism that takes greater than 3 weeks to demonstrate a measurable response.

Vertigo is often cited as a poor prognostic indicator for sudden hearing loss mostly due to large reviews performed prior to ITS in the 1970s and 1980s [15, 21, 22]. Most recent studies involving ITS have reported that vertigo is not a significant prognostic indicator [5–7, 13, 23] while reports of vertigo as a negative prognostic indicator are the minority [24]. We found a significantly higher rate of response to ITS in patients who presented with vertigo. It has been our experience that when patients experience vertigo with their sudden hearing loss, they are much more likely to seek prompt medical evaluation and are more likely to be referred to a tertiary referral center. We believe that it is the shorter interval between hearing loss and the start of treatment in patients with vertigo (18 days to treatment) versus those who did not (32 days to treatment) that best explains this result in our series.

Like other reviews, our data are limited by not having serial audiograms, which would better define when our patients responded to treatment. It is possible that the patients in our group who had audiograms >5 weeks after the first dose of ITS responded sooner, which would have been detected if their audiograms had been performed earlier. However, our data differ from the currently reported data in that they show recovery occurring late, rather than early. In addition, our study highlights an area of ITS that is still yet to be defined: the timing of post-treatment audiograms in assessing treatment response.

## 5. Conclusion

Our observed response of a significant number of patients showing improvement when audiograms were performed >5 weeks after the first dose of ITS is contrary to the idea that response to treatment occurs early after starting treatment. This may reflect a group of patients with delayed response to steroid treatment. To better define when to perform post treatment audiograms and to attempt to stratify patients with ISSNHL as early or late responders, studies should report when audiograms are administered and should ideally have multiple audiograms to monitor response.

## Figures and Tables

**Table 1:** Inclusion and exclusion Criteria.

30 dB loss in 3 consecutive frequencies in <72 hours	
Pre- and post-treatment audiogram including PTA and SDS	
Normal or near normal hearing in the contralateral ear normal otoscopic exam	
Normal MRI with contrast of the brain and internal auditory canals	
Negative serologic studies for infectious and inflammatory disease	
No history of chronic otitis media	
No history of trauma (head, acoustic, or barometric)	
No history of Meniere's disease, hydrops, or fluctuating hearing loss	
No history of meningitis	
No history of prior ear surgery	
No history of radiation	
No exposure to ototoxic mediations	

dB: decibel; PTA: pure tone average; SDS: speech discrimination score.

**Table 2:** Demographic characteristics of the study population.

Age (years)	
Mean	54
Range	13–84
Sex	
Male	20
Female	20
Ear	
Right	14
Left	26
Oral steroids prior to ITS	25
Days to treatment with ITS	
Mean	22.7
Range	1–120
≤14	15
15–28	12
>28	13

ITS: intratympanic steroids.

**Table 3:** Average pre- and post-treatment PTA and SDS.

	Pre		Post		Diff^†^		
	Mean	SD	Mean	SD	Mean	SD	*P*-value*
PTA, dB	73	24	56	27	−17	21	<0.001
SDS, %	37	34	49	38	12	30	0.02

^†^Difference is post-pre.

*P-values were calculated with a paired *t*-test.

PTA: pure tone average; SDS: speech discrimination score; dB: decibel.

**Table 4:** Patient variables and associated response to treatment.

	Overall (*N* = 40)		Improvement (*N* = 18)		No Improvement (*N* = 22)		
	*N*	%	*N*	%	*N*	%	*P*-value*
Time to post-ITS audiogram							
≤5 weeks	27	68	9	33	18	67	0.03
>5 weeks	13	33	9	69	4	31	
Age, years							
Mean (SD)	54	(18)	50	(18)	58	(18)	0.17
Gender							
Male	20	50	8	40	12	60	0.53
Female	20	50	10	50	10	50	
Ear							
Left	26	65	10	38	16	62	0.26
Right	14	35	8	57	6	43	
Days from onset to ITS							
≤21	24	60	12	50	12	50	0.02
>21	16	40	6	38	10	63	
Prior oral steroids							
Yes	25	63	9	50	9	50	0.14
No	15	38	9	36	16	64	
Vertigo							
Yes	17	43	11	65	6	35	0.03
No	23	58	7	30	16	70	
Tinnitus							
Yes	27	68	14	52	13	48	0.21
No	13	33	4	31	9	69	

*P-values were computed using *t*-test for continuous characteristics and chi-square tests for categorical. ITS: intratympanic steroids; *N*: frequency; SD: standard deviation.

**Table 5:** Adjusted odds of improvement (*N* = 40).

		OR	95% CI	*P*-value
Weeks to post-ITS audiogram	>5 weeks versus ≤ 5	6.7	(1.3, 35)	0.02
Vertigo	Yes versus No	6.2	(1.3, 29)	0.02

OR: odds ratio; CI: confidence interval; ITS: intratympanic steroids.
